# Case Report of a 4-Year-Old Girl with Stage IV Grade C Localized Periodontitis (Pre-Puberal Localized Aggressive Periodontitis) Affected by Misrecognition and Late Diagnosis

**DOI:** 10.3390/jcm13010266

**Published:** 2024-01-03

**Authors:** Radu-Andrei Moga, Cristian Doru Olteanu, Ada Gabriela Delean

**Affiliations:** 1 Department of Cariology, Endodontics and Oral Pathology, School of Dental Medicine, University of Medicine and Pharmacy Iuliu Hatieganu, Str. Motilor 33, 400001 Cluj-Napoca, Romania; ada.delean@umfcluj.ro; 2 Department of Orthodontics, School of Dental Medicine, University of Medicine and Pharmacy Iuliu Hatieganu, Cluj-Napoca, Str. Avram Iancu 31, 400083 Cluj-Napoca, Romania

**Keywords:** juvenile aggressive periodontitis, periodontal pocket, bone loss, hypophosphatasia, diagnosis, temporary dentition

## Abstract

Background and Objectives: Stage IV grade C localized periodontitis (pre-puberal localized aggressive periodontitis/LPP) is a rare form of inflammatory periodontal disease occurring in clinically healthy individuals (no/small calculus/dental plaque traces), due a hyper-aggressive auto-immune response to high amounts of bacteria present in the oral cavity. Case Presentation: This case report describes a 4-year-old Caucasian girl with localized gingival inflammation and advanced bone loss around the temporary lower left canine. The first diagnostic assumption was hypophosphatasia, and the patient was sent for further genetic and metabolic investigations (which turned out to be negative). The LPP diagnosis was made during the family’s summer holidays due to her parents’ concerns about persistent gingival inflammation and tooth mobility. Results: The diagnosis of LPP was supported by clinical oral examination results, earlier X-rays, earlier blood tests, and a periodontal bacterial test. The treatment was limited to avoid spreading inflammation to other teeth (via topical antibiotic treatment) due to our limited time frame, while the main problem of excessive amounts of periodontal bacteria in the oral cavity was not addressed. The tooth was eventually lost. Conclusions: The ability to early recognize radiological and clinical LPP signs correlated with understanding of its pathological auto-immune mechanism is extremely important for expanding treatment options, since bone preservation and reducing amounts of bacteria are strictly correlated with therapeutic speed.

## 1. Introduction

Stage IV grade C localized periodontitis (formerly known as localized aggressive periodontitis (LAP)/localized juvenile periodontitis) is a rare form of inflammatory periodontal disease occurring in clinically healthy individuals (with a 0.06% prevalence in European white individuals and an up to 2.6% prevalence in African Americans) [1,2,3,4,5]. Early-age onset and rapid progressive bone and periodontal ligament (PDL) loss around very specific teeth (first, the molars and incisors) correlated with low/no calculus or oral plaque deposits and surrounding periodontal tissues with almost no inflamed aspects upon clinical inspection are some of the main pathognomonic signs [1,2,4,5,6,7,8,9,10]. One radiological characteristic is deep “U”-shaped bone loss [1,6].

This rare form of periodontitis has been reported to show familial aggregation (i.e., hyperinflammatory auto-immune response when encountering periodontopathic bacteria in blood samples) [2,3,4,11]. LAP is a multifactorial disease, with its leading role being abnormal response to microbial communities present in the oral cavity [3]. In healthy individuals, there is a symbiotic relationship between bacterial community and oral biofilm [3]. However, if a genetic predisposition and/or environmental factors (not yet characterized) are met, the normal symbiotic relationship becomes abnormal and dysbiotic, producing an extremely aggressive auto-immune response leading to tissue destruction due to disruptions in bone metabolism [2,3]. 

Stage IV grade C localized periodontitis (former known LAP) may involve both primary (pre-puberal periodontitis LPP, affecting more first molars) and permanent (juvenile periodontitis LPS, involving more first molars and incisors) dentition (genetic predisposition) [1,10]. 

Periodontal disease (of which LPP is a part) has plaque biofilm as its primary agent (i.e., bacterial communities embedded in extracellular polymeric substances), with increased resistance to antimicrobial agents and immune defenses compared with planktonic counterparts [12]. The final phase of biofilm development is the detachment of cells, where bacteria disperse as free planktonic bacteria. Moreover, the dispersed bacteria have a higher virulence (e.g., Pseudomonas aeruginosa) [12]. However, there is no general universal mechanism of biofilm dispersal; local conditions (increased/reduced availability of certain substances) enhance the virulence and number of certain bacteria [12,13]. The main bacteria involved in periodontal disease are *Fusobacterium nucleatum*, *Porphyromonas gingivalis*, *Treponema denticola*, and *Tannerella forsythia* [12,13]. It has been reported that in the absence of *Fusobacterium nucleatum*, the number of late colonizers associated with periodontal destruction is significantly reduced [12,13].

Reports of LPP patients have shown a higher number of lymphocytes (particularly B cells) within the immune response [2,14], correlated with low amounts of plaque deposits, hyper-responsive macrophage phenotypes, and *Actinobacillus actinomycetemcomitans* and *Porphyromonas gingivalis* [10,13]. A recent study reported that *Actinobacillus actinomycetemcomitans* triggers the chemokine response by secreting ATP (adenosine triphosphate), an important intercellular molecule secreted by activated immune cells and/or damaged cells which alerts the immune system to presumed imminent danger [13]. Moreover, in secreting the APT molecule, bacteria has been reported to be highly virulent and a major contributor to inflammation during periodontal disease [13]. The same study reported *Porphyromonas gingivalis*, *Fusobacterioum nucleatum*, and *Prevotela intermedia* to not secrete the ATP molecule [13]. Nevertheless, in the subgingival environment where these bacteria are met, there are constant changes in nutrients, oxidative stress due to mechanical plaque disruption, and ATP molecules, conditions that are exceedingly difficult to study and completely understand [12,13]. 

More severe gingivitis increasing with age, possibly due to changes in plaque levels, bacteria composition, hormonal changes, inflammatory response, and tooth eruption, has also been reported [1]. Tissular periodontal destruction occurs in the situation of an immunological imbalanced response of the organism to the presence of sub-gingival bacterial (requiring bacterial-type tests) [2]. The auto-immune response to the presence of bacteria is a rapid and severe inflammatory response with infiltration of immune cells, resulting in tissue reportion [2,11,14]. Nevertheless, there are contradictory data related to the relationship between the response to certain bacterial types and the exaggerated immune response to any bacteria [2,11,15]. In the presence of genetic predisposition and/or environmental factors (presently unknown), the immune response becomes excessively aggressive (i.e., activating an intracellular signaling cascade with inflammatory cytokines, P2Rx7 functional diplotypes, chemokines, adhesion molecules, and growth factors) ending with tissular destruction [2,3,11,15]. 

Periodontitis can be predictably treated in the initial stages (stressing the importance of disease recognition) [5]. The overall treatment of and goals for LAP and chronic periodontitis patients are similar (systemic initial diagnosis, re-evaluation, and surgical management, followed by maintenance and restorative phases); it is important to control local and systemic risk factors, manage inflammation, and arrest disease progression [6,10,16,17,18,19]. Treating LPP patients is challenging due to the rapid progression of disease and the smaller periodontal attachment area when compared with permanent teeth [5]. The aim is to stop periodontal loss (both of PDL and bone) to regain as much as possible by establishing a healthy periodontium, to retain as many teeth and for as long as possible, and to avoid/minimize the spread of the condition to permanent teeth [5,6,10,17].

Nonsurgical therapy (i.e., scaling and root planning) is reported to achieve periodontal stability in up 3 to 6 months, but with reports of relapse after this period of time, despite frequent recall visits and oral hygiene reinforcement (showing a less predictable response to conventional therapy than chronic periodontitis) [5,6,9,10,17,18,19]. 

Systemic antibiotics (e.g., metronidazole and amoxicillin combinations, which are the most commonly used) correlated with nonsurgical therapy seem to supply better results, since bacteria invading subepithelial tissues are difficult to eradicate with scaling and root planning alone [5,6,9,10,16,17,18]. Local antimicrobials and antibiotics have been less investigated in correlation with non-surgical therapy, but there are reports of a combination of amoxicillin and metronidazole playing a positive role in the treatment of localized forms [5,6,9,10,16,19]. There is limited evidence reported for scaling and root planning associated with extraction of affected teeth to avoid the spread of periodontal disease to permanent teeth [5]. Other less-used non-surgical treatments include laser/photodynamic therapy and host response modulation [6,9]. The concept of “full-mouth disinfection” within 24 h (the association of scaling and root planning with antibiotics and disinfectants) has been shown to improve clinical outcomes [6,7].

Surgical therapy provides direct access to the root surface and furcation areas with better debridement and the possibility of bone recontouring and/or regenerative techniques, with few studies specifically addressing aggressive periodontitis, but with positive results [6,10]. Nevertheless, for young children, surgical therapy is usually not an option.

In this paper, the case of a 4-year-old Caucasian girl (with no identified family history) with Stage IV grade C localized periodontitis (LPP) of the temporary lower left canine (7.3) is described. Additionally, related clinical investigations are presented and discussed to emphasize the medical reasoning that led to diagnosis. Despite clinical evidence and paraclinical investigations, the patient was examined by multiple general dentists and periodontists, and misinterpretation of objective symptoms and paraclinical investigation lead to an incorrect course of treatment (i.e., her initial diagnosis was of hypophosphatasia/hyperphosphatasia [20,21]), with a late correct LPP diagnosis and start of treatment. Unfortunately, due to this long history, the tooth and surrounding periodontium was lost.

Hypophosphatasia is an extremely rare bone metabolism disorder (with a very low prevalence of 1 to 300,000 births in Europe) caused by deficiency in alkaline phosphatase activity and ALPL gene mutation, involving calcium and phosphate metabolism [20]. There are two main forms: odonto-hypophosphatasia and systemic hypophosphatasia (with five forms) [20,21]. It is characterized by defective bone mineralization due to a nonspecific isoenzyme of alkaline phosphatase, since the absorption of calcium is normal, but there is lack of fixation in bones [20,21]. In addition, hypophosphatasia patients have high phosphate serum levels (hyperphosphatemia) due to increased renal tubular phosphate reabsorption [21]. Odonto-hypophosphatasia is manifested through dental abnormalities, premature loss of deciduous teeth, severe dental caries, reduced dentine thickness, enlarged pulp chamber, reduced alveolar bone height, absence of associated musculoskeletal abnormalities, and other abnormalities [20]. The blood tests show low levels of parathyroid hormone due to hypercalcemia and hypercalciuria, which would lead to the development of hyperphosphatasemia [20,21]. We must emphasize that none of these results were seen either clinically or on the initial blood test results.

## 2. Materials and Methods

### 2.1. Case Presentation

In the spring of 2023 (April–May), a Caucasian girl of four years and three months was referred to her local family dentist (in a small suburb of München, Germany) for a regular checkup due to frequent complains of small-to-moderate oral pains with variable localization and intensity. The parents were informed by the professional that upon intra-oral clinical examination, nothing abnormal was found, and the pain complaints were of no obvious cause.

The pain complaints continued, and in June, the small girl told her parents that her lower left temporary canine (i.e., 7.3) began to move. The parents returned to the local dentist, who saw the abnormality of the case (temporary canine mobility so early) and oriented the girl to the München University Hospital (Periodontology Department).

**Figure 1:**
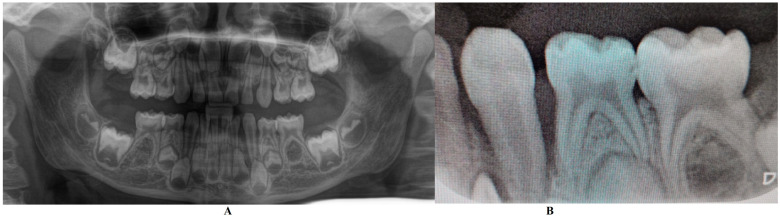
X-rays taken in the Periodontology Department of München University Hospital (early June): (**A**)—panoramic with localized bone loss around temporary lower left canine, (**B**)—retro-alveolar detailed X-ray of the same area.

In the Periodontology Department (14 June), a full investigation was performed including one panoramic and retro-alveolar radiological examination (Figure 1). This radiological examination confirmed the localized nature of the bone loss with no visible signs of other disorders. The written results were pains of unclear origin, early temporary canine and bone loss, all caused by a possible metabolic disease (hypophosphatasia/hyperphosphatasia), with the recommendation to follow the metabolic–endocrinology path. The patient was sent to the Endocrinology Department for blood and genetic tests. No clinical signs of orthodontic problems (e.g., hyper-eruption) were found at this stage. No indication of treatment was given to the parents, except recall (i.e., after genetic and metabolic tests results) and prophylaxis (common oral hygiene).

In the Endocrinology/Metabolic Disease Department (19 June), blood test samples were taken. The blood analysis results reported only a slight increase in monocytes and lymphocytes, and a small decrease in neutrophile granulocytes (20 June). The urine test was negative (21 June). No endocrine or genetic disorders were detected (17 July—received in late September by the parents), confirming the initial suspicion of hypophosphatasia.

**Figure 2:**
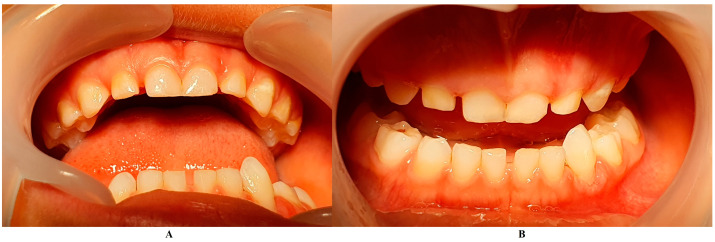
(**A**,**B**) Intra-oral cavity general aspect (in early August) with the localized temporary canine problem that led to the LPP diagnosis.

**Figure 3:**
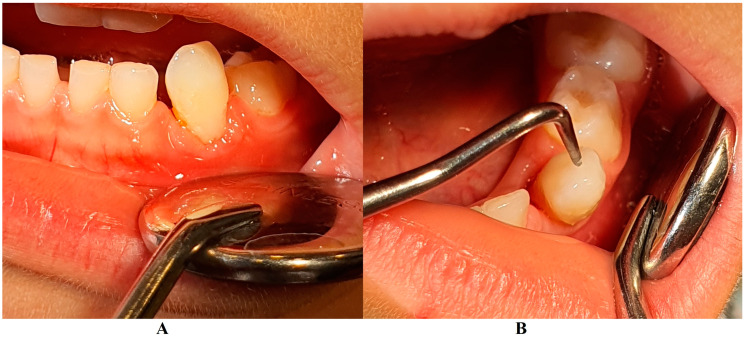
(**A**)—Gingival inflammation, (**B**)—advanced tooth mobility, which led to the LPP diagnosis (details, in early August).

**Figure 4:**
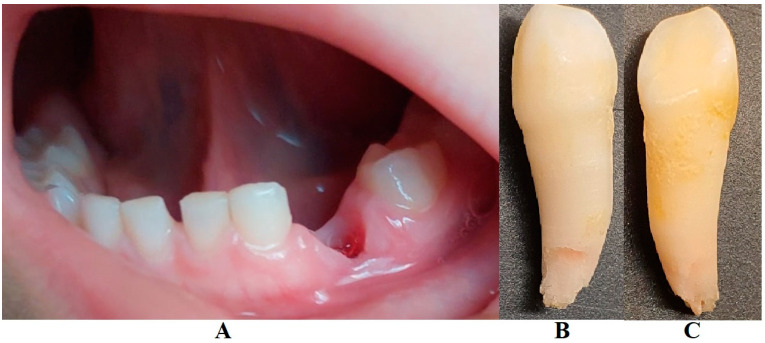
Tooth loss (September): (**A**)—alveolar socket with no inflammation signs, (**B**,**C**)—tooth with root apical third resorptive signs and traces of calculus and dental plaque in the root cervical area and middle third (the potential origin of the periodontal problems).

The family spent the summer holidays in Klausenburg/Cluj-Napoca (Romania), where they consulted another dentist (2 August), since tooth movements increased, and they were concerned about the outcome. After the clinical examination and review of previously available radiographs, blood tests, and the patient’s clinical history, a diagnosis of Stage IV grade C localized periodontitis/LPP was established (hypophosphatasia/hyperphosphatasia was excluded from the beginning, despite the fact that genetic test results were not yet available) (Figure 2 and Figure 3). These two figures show the case in early August, with a localized hyper-eruption of the tooth due to extended periodontal loss, inflammation in periodontal pocket, and hyper-immune response. We must emphasize that till early August, no visible signs of orthodontic problems (e.g., hyper-eruption) were found after multiple clinical examinations. The earlier X-ray examination (from early June, Figure 1) was taken into consideration when the diagnosis of Stage IV grade C localized periodontitis/LPP was formulated. To confirm the laboratory diagnosis and identify the type of bacteria involved, a periodontal pocket bacterial test sample was taken (laboratory confirmation, 3 August), and in the meantime, professional oral hygiene and an adjuvant topical combination of amoxicillin and metronidazole was applied for a period of 10 days (due to advanced surrounding bone loss). The periodontal bacteria pocket test came positive, with increased levels of *Fusobacterium nucleatum/periodonticum* and *Capnocytophaga* spp. (16 August) confirming the Stage IV grade C localized periodontitis/LPP diagnosis. Because the tooth movement was significant by the time of LPP diagnosis due to advanced periodontal support loss, the tooth was lost at the beginning of September (Figure 4).

### 2.2. Differential Diagnosis and Genetic, Urine, Blood, and Periodontal Pocket Bacterial Tests

The first diagnosis (of presumed hypophosphatasia/hyperphosphatasia) was established in the Periodontology Department of München University Hospital in the middle of June (14 June), despite the available radiographical examination acquired during the full clinical examination (Figure 1) and patient’s history (with no visible signs of disorder). The radiographical examination clearly showed a massive, localized bone loss around the temporary lower left canine (i.e., 7.3—Figure 1B) visible on the retro-alveolar X-ray, with no other visible radiological signs present upon panoramic X-ray (Figure 1A). During clinical examination, increased tooth movements were also present, with no other clinical signs (at least, after the parents’ report). Moreover, blood tests (19 June) showed normal levels of calcium, phosphate, natrium, and parathyroid hormone, and absence of hypercalciuria, which contradicts the hypophosphatasia/hyperphosphatasia diagnosis [20]. No other diagnosis or treatment approach was performed.

On the other hand, when the first Stage IV grade C localized periodontitis/LPP diagnosis was established (2 August), the clinical examination showed an increased mobility of the temporary lower left canine due to massive bone and periodontal ligament loss, associated with a localized inflammation of the free gingival margin around the tooth, and with small amounts of dental plaque due to difficulties with oral hygiene (Figure 2 and Figure 3). No other plaque deposits, inflamed gingiva, or teeth mobility issues were detected during the clinical examination. When this information was correlated with the radiological examination from two months prior (19 June, Figure 1), with localized bone loss, the diagnosis based on clinical and radiological data was confirmed, with no need for further radiological examination. Moreover, the blood test with higher levels of lymphocytes [2,14] also confirmed Stage IV grade C localized periodontitis/LPP diagnosis. To assess the periodontal pocket bacteria responsible for this atypical LPP case (i.e., temporary molars/incisors are usually involved [1,2,10], rather than the canine, as in this case), a canine periodontal sulcus secretion test was sampled and sent to the laboratory.

### 2.3. Dental and Periodontal Management

Following the extremely late Stage IV grade C localized periodontitis/LPP diagnosis, the therapeutic approach consisted of reducing the number of bacteria present in the canine periodontal pocket to identify the cause of the problems, to avoid the spread of infection to the other teeth, and to maintain proper oral hygiene while informing the parents about the outcome of canine loss (eventually, to be lost in a few weeks) and the prognosis of the case (with the possibility of more problems).

Periodontal pocket fluid was sampled (using sterile paper cones from the periodontics special kit) and sent to the laboratory to identify bacterium types.

The therapeutic approach was limited to a proper professional cleaning of the canine and administration of a topical solution (three times a day) of amoxicillin and metronidazole in the periodontal pocket, since the family was leaving town and patient was to be under no medical supervision (despite knowing that systemic antibiotic treatment would be certainly more efficient). After two days, gingival inflammation was significantly reduced (as expected), but tooth mobility was also guarded (due to advanced bone loss).

After two weeks (16 August), the periodontal test came back positive for *Fusobacterium nucleatum/periodonticum* and *Capnocytophaga* spp., confirming both the Stage IV grade C localized periodontitis/LPP diagnosis and the initial choice of antibiotics (according to Deutsche Gesellschaft für Zahn-, Mund- und Kieferheilkunde, and other scientific reports [10,16]).

Eventually, as expected, the tooth was lost at the beginning of September, and alveola healed without further problems (Figure 4).

## 3. Discussion

This unusual case of Stage IV grade C localized periodontitis/LPP demonstrates how a case can go misrecognized and undiagnosed for a long period of time, with severe consequences for the patient and their family if radiological and clinical signs are missed (despite the large window of opportunity within which to take a therapeutic approach [5,6,9,10,16,17,18,19,22]). Since LPP is a rapidly progressing disorder, lack of treatment for a few months (i.e., four to five months, as reported here) could lead to periodontal support and temporary tooth loss, with further consequences (i.e., orthodontic and periodontic) [1,2,5,10].

A rapid Stage IV grade C localized periodontitis/LPP diagnosis is essential, since the aggressive auto-immune response to bacteria can rapidly lead to a resorptive process [1,2,3,4,5,6,7,8,9,11]. Thus, a simple bacterial test to identify the pathogenic bacterium types would provide both diagnostic confirmation and justification for antibiotic treatment [2,3,11,12,13,14,15]. If LPP disorder is identified early, there is time to wait (i.e., usually up to two weeks) for lab test results, and only then should antibiotic therapy proceed [5,6,9,10,16,17,18]. However, if the LPP diagnosis is late (as it was herein), antibiotic treatment must be started, with a combination of two large-spectrum antibiotics (amoxicillin and metronidazole) to cover most bacterial types [5,6,9,10,16,17,18]. It must be emphasized that LPP usually comes without or with very few plaque deposits (as the case herein showed—see Figure 1, Figure 2, Figure 3 and Figure 4), with little influence over surrounding gingival inflammation [1,2,4,5,6,7,8,9,10].

Due to the hyper-aggressive auto-immune response to periodontal bacteria [2,3,11,14,15] present in the temporary canine periodontal pocket and a lack of visible calculus and plaque, the therapeutic approach focused on reducing the amount of bacteria to diminish the immunological response and thus the aggressive manifestations. We must emphasize that systemic antibiotic treatment was seen as the better approach. However, since the family stayed only for a brief period of time, the 4-year-old child would then be under no medical supervision during a potential systemic antibiotic treatment (the family wished to take no risks). Thus, the only remaining available option was topical treatment, which diminished periodontal inflammation.

The management of this LPP case focused on pursuing the periodontal treatment goals of conserving as much bone as possible and avoiding the spread of inflammation/infection to other teeth [5,6,9,10,17,18,19]. The temporary canine was considered too compromised to be saved due to the massive periodontal loss. Nevertheless, the main source of bacteria was the periodontal pocket; thus, the topical antibiotic treatment focused on this area. However, due to the limited time frame for treatment, therapeutic possibilities were limited, despite having both means and knowledge.

The bacterium types present in this case (i.e., *Fusobacterium nucleatum/periodonticum* and *Capnocytophaga* spp.) were reported not to produce ATP molecules [13]; nevertheless, the presence of *Fusobacterium nucleatum* has been reported to be associated with a high number of late colonizers associated with periodontal destruction [12,13], which might explain some of the localized aggressive periodontal destruction. Nevertheless, on the lost tooth (Figure 4B,C), some traces of calculus and plaque are visible, confirming the source of periodontal bacteria.

The patient had no earlier known medical history of Stage IV grade C localized periodontitis/LPP or LAP to explain the aggressiveness of oral bacteria [2,3,4,11]. No explanation for the cause, moment or tooth could be identified. However, the familial history includes periodontal disease, cancers, and other degenerative diseases (providing a possible explanation for their auto-immune hyper-response, as a clinical assumption).

The first approach to this case (München University Periodontology Department—blood, urine, genetic, metabolic, and endocrine tests) was clinically practical, since no immediate danger was visible, no other inflammation signs were seen, no plaque and calculus were present, and the tooth already showed massive periodontal destruction, and high mobility, and would eventually be lost. The only aspect that is unsettled is the initial hypophosphatasia/hyperphosphatasia assumption despite no clinical or radiological signs being present [20,21]. However, since this disease relies on a hyper-aggressive auto-immune response to bacteria (when amounts of bacteria are high), in the medium and long term, the danger of disease spreading to other teeth was not negligeable, and disease was expected to aggravate. Thus, from a periodontal clinical conservative point of view, controlling the amounts of bacteria was also a viable option, and was associated with identifying the bacterium types present in the periodontal pocket [5,6,9,10,17,18,19].

Retrospectively speaking, the tooth showed visible traces of plaque and calculus deposits (Figure 4B,C), which could be present on the tooth throughout the infectious episode, going unnoticed, and representing the original source of bacteria. Nevertheless, it must be emphasized that full professional scaling and root planning was extremely difficult in this case, due to the child’s small age and problems of collaboration (hence the importance of individualizing periodontal treatment).

In this clinical case, due to the long clinical history and the limited period of time between LPP diagnosis and the family leaving town, the window of opportunity was missed, in spite of an immediate reduction in aggravation risks due to prophylactic measures (i.e., hygiene and topical antibiotic therapy). However, the main problem of the considerable amounts of bacteria in the oral cavity (confirmed by the bacterial test results, which arrived after the family left town) remained unsettled. Further risks in the medium and long term of disease spreading to other teeth due to bacteria multiplication were expected to be significant, since no active measures to reduce the amounts of bacteria present in the oral cavity were taken. Hygiene alone is not enough, since LPP is a disorder with no/reduced plaque deposits; the tissular destruction is due to a hyper-aggressive auto-immune response. There is a need for constant clinical and radiological monitoring of the child, and when visible inflammation signs appear, rapid active measures (including systemic antibiotic therapy based on bacteria tests) need to be taken. These issues were communicated to the parents, remaining to be further addressed in their hometown.

The case herein proves the importance of paying attention to little children’s complaints of pain and investigating them if they are frequent [22]. A simple digital panoramic or retro-alveolar radiographical examination (i.e., without any risks for patients’ health) would produce further data which would help to identify any potential problem [22].

If localized bone loss is detected, this usually indicates a localized disorder due to high levels of bacteria in the oral cavity, and not a general systemic problem, which usually manifests in the entire oral cavity. The panoramic X-ray provides general data about the bone levels not only around teeth but also in both bone maxillary structures, with an orientation versus LPP and exclusion of hypophosphatasia [20,21].

Knowing and understanding the pathological mechanism behind the oral manifestation of LPP and LAP helps in exploiting the window of opportunity for avoiding periodontal loss; if necessary, measures can be taken immediately.

Blood tests usually help, especially if they show normal values/intervals; any increased levels can confirm or orientate the diagnosis [2,14]. In the case herein, higher level of lymphocytes [2,14] and normal levels of calcium and phosphates, parathyroid hormone, and urine [20,21] would support the assumption of LPP and would rapidly exclude hypophosphatasia, along with the clinical examination results.

Diagnostic errors are a known problem in clinical practice, but there are few data available regarding this issue [22]. Thus, the case report herein focused on reporting a problem, and on providing medical education showing and arguing the medical reasoning and clinical evidence for avoiding further similar problems. Clinical cases, even if unusual (as herein), must be approached with careful reasoning, aiming to follow the periodontic treatment goals of conserving the periodontium and keeping as many of the teeth in the oral cavity as possible (i.e., if possible), especially in cases involving small children, thus avoiding further orthodontic and periodontic disorders [10,16].

## 4. Conclusions

This rare case of Stage IV grade C localized periodontitis/LPP emphasizes the need to know and understand its pathological mechanism for early interception and diagnosis, in order to minimize periodontal loss. Only in understanding these issues can proper medical reasoning be reached, and practical personalized periodontal treatment given. The treatment must take into consideration not only short- but also medium- and long-term risks for the young patient. The ability to recognize early radiological and intra oral cavity signs and correlate them with their most probable cause will allow us to gain back some time for tests that can confirm diagnosis and allow a more appropriate therapeutic approach. However, if diagnosis is late, in addition to the problem of advanced tissue loss, the timeframe for tests and medical reasoning becomes extremely small, all to the detriment of the patient.

## Data Availability

No new data were created or analyzed in this study. Data sharing is not applicable to this article.
